# Virus Injection to the Pituitary *via* Transsphenoidal Approach and the Innervation of Anterior and Posterior Pituitary of Rat

**DOI:** 10.3389/fendo.2020.546350

**Published:** 2020-12-04

**Authors:** Xiaohui Li, Shanchun Su, Haiwen Zhao, Yang Li, Xueqin Xu, Yan Gao, Dongsheng Sun, Zeyong Yang, Weilin Jin, Changbin Ke

**Affiliations:** ^1^ Institute of Anesthesiology and Pain (IAP), Department of Anesthesiology, Hubei Key Laboratory of Embryonic Stem Cell Research, Hubei Key Laboratory of Wudang Local Chinese Medicine Research, Taihe Hospital, Hubei University of Medicine, Shiyan, China; ^2^ Department of Anesthesiology, International Peace Maternity and Child Health Hospital, Shanghai JiaoTong University School of Medicine, Shanghai, China; ^3^ Institute of Nano Biomedicine and Engineering, Department of Instrument Science and Engineering, Key Lab. for Thin Film and Microfabrication Technology of Ministry of Education, School of Electronic Information and Electronic Engineering, Shanghai JiaoTong University, Shanghai, China

**Keywords:** rat, anterior pituitary, posterior pituitary, innervation, pseudorabies virus

## Abstract

The theory holds that the anterior pituitary in mammals receives humoral regulation. Previous studies have reported that the pars distalis of the anterior pituitary of several mammalian species contains substance P-, calcitonin gene-related peptide (CGRP)-, and galanin-like immunoreactive nerve fibers, but the origins of these nerve fibers are unclear. Removal of the pituitary gland, also called hypophysectomy, involves methods that access the pituitary gland *via* the transauricular or parapharyngeal pathways. However, these methods are not applicable for viral tracer injection to investigate the innervation of the anterior pituitary. The transauricular technique leads to inaccuracies in locating the pituitary gland, while the parapharyngeal approach causes high mortality in animals. Here, we introduce a protocol that accesses the pituitary gland in the rat *via* the transsphenoidal pathway. This method imitates surgical manipulations such as endotracheal intubation and sphenoid bone drilling, which involve the use of custom-made devices. Using the transsphenoidal pathway greatly improves the survival rate of rats because no additional dissection of blood vessels and nerves is required. Moreover, the pituitary gland can be viewed clearly and directly during the operation, making it possible to accurately inject pseudorabies virus (PRV) 152-expressing enhanced green fluorescent protein (EGFP) into the anterior or posterior pituitary, respectively. After injecting PRV 152 into the anterior pituitary, we found no evidence of direct innervation of the anterior pituitary in the rat brain. However, PRV 152 injection into the posterior pituitary revealed retrograde transneuronal cell bodies in many brain areas, including the CA1 field of the hippocampus, the basolateral amygdaloid nucleus, posterior part (BLP), the arcuate hypothalamic nucleus (Arc), the dorsal portion of the dorsomedial hypothalamic nucleus (DMD), the suprachiasmatic nucleus (SCh), and the subfornical organ (SFO). In the present study, we provide a description of a possible model of hypophysectomy or pituitary injection, and identify brain regions involved in regulating the rat pituitary gland using transneuronal retrograde cell body labeling with PRV.

## Introduction

The pituitary gland, also called the master gland, is a fundamental regulator of the endocrine system, with its activity leading to complex metabolic events and affecting a number of physiological processes, including growth, lactation, and stress (1). Therefore, it is essential to comprehensively understand the role and function of the pituitary gland in the endocrine network and the central nervous system (CNS).

The pituitary gland is divided into the anterior and posterior lobes. Only a few autonomic nerve fibers determine the vasoconstriction of gland cells in the anterior lobe, while the secretory activity of gland cells involves no direct nerve innervation and is instead essentially regulated by a variety of hormones from the hypothalamus. The conventional wisdom has been challenged on the basis of much research attention over many years. Payette et al. found serotonin (5-HT) immunoreactivity in endocrine cells of the anterior lobes of the pituitary glands in mice and bats using light and electron microscopy combined with immunocytochemistry (2). Moreover, Mikkelsen et al. demonstrated that a number of substance P (SP)-like immunoreactive (LI) nerve fibers distributed around pituitary cells. They revealed *via* kinetic analysis that the anterior pituitary contains specific binding sites for SP (3). Furthermore, Skofitsch and Jacobowitz detected a number of CGRP-LI nerve fibers in the anterior pituitary of the rat (4). Ju et al. reported that the peptidergic fibers in the anterior pituitary are closely associated with the glandular cells *via* synaptic contacts, meaning that the activities of the anterior pituitary are probably regulated by neural factors (5). Ju proposed the hypothesis that the mammalian anterior pituitary receives neural–humoral dual regulation (6). From these studies, it is obvious that the regulation and function of anterior pituitary are complex and deserve to be studied in greater depth. At present, the origins of the nerve fibers innervating the anterior pituitary are unclear, and it remains to be determined whether these nerve fibers come from the hypothalamus, the peripheral nervous system, or both. Therefore, this makes it particularly important to explore the innervation of anterior pituitary using viral tracer injection.

The pituitary gland is situated at the base of the brain in the rat and is protected by a bony structure called the sella turcica of the sphenoid bone (7), an anatomical feature that is maintained throughout the entire lifetime of the rat. Surgical interventions, such as transauricular (8, 9) or parapharyngeal (10, 11) techniques, have been applied to explore the role of the pituitary gland in endocrine and CNS function. Previous descriptions of surgical techniques for exposing the pituitary or for performing hypophysectomy in the rat are decades old. The transauricular surgical technique is relatively convenient, but it cannot be relied upon to accurately locate the pituitary gland. The parapharyngeal surgical approach is rather complicated, requiring a greater number of nerves and blood vessels to be dissected. Here, we describe a modified approach based on the transsphenoidal hypophysectomy technique that has been used in dogs for many years, which reaches the basisphenoid *via* the retropharyngeal route (12–14). Our technique approaches the pituitary gland *via* the oral cavity after exciding the soft palate and avoids severe damage to blood vessels, nerves, and other tissues. Furthermore, in the rat, the pituitary gland is located on an almost flat sheet of tissue at the base of the brain, unlike in other animals, in which it is located within the most inferior aspect of the sella turcica. We can easily identify the exact location of the pituitary based on the local anatomical features. The major procedures described in this method include anesthesia, fixation in the supine position, endotracheal intubation, soft palate incision, sphenoid bone drilling, and injection of the virus. In the present study, AAV2/Retro and PRV 152 were separately injected into the anterior and posterior pituitary to investigate the innervation of these brain regions. Injection of AAV2/Retro or PRV 152 into the anterior pituitary showed no evidence of any direct innervation. However, following injection of PRV 152 into the posterior pituitary, retrograde transneuronal cell bodies were detected in many brain regions after 4 days, including hippocampal CA1, BLP, Arc, DMD, SCh, and SFO.

In the present study, we present a reliable technique for discerning the exact location of the pituitary gland and provide experimental details to guide the accurate injection of this structure. Furthermore, using transneuronal retrograde cell body labeling, we identify the brain regions in the CNS that innervate the pituitary gland in the rat. The surgical approach we use to expose the pituitary is applicable both to hypophysectomy and pituitary injection, and it provides a unique platform to explore the functional significance of the pituitary gland in the endocrine system and in the CNS.

## Materials and Methods

### Animals

All experiments acceptable to this study were examined and approved by the Animal Care and Use Committee of Hubei University of Medicine (Hubei, China). Male Sprague Dawley (SD) rats weighing 220–250 g were obtained from the Institute of Laboratory Animal Science, Hubei University of Medicine. Animals were maintained in controlled conditions for 12 h alternating light/dark cycle, food and water *ad libitum*. A total of 42 rats were used in this study to perform virus injection in the anterior pituitary and posterior pituitary.

### Preparation for Surgery

The implements or accessories used in the study included suction apparatus, electrotome, dental drill, surgical microscope, stereotactic apparatus, custom-made elastic retractors, bone wax, and surgical suture. The surgical instruments were sterilized with 70% ethanol and dried on a paper towel. Animals were anesthetized following intraperitoneal (i.p.) injection of pentobarbital sodium (35 mg/kg) and supervised every 2 min by the response to toe pinch with forceps to check for appropriate depth of anesthesia. No startle reflex to the pinch indicated the animals were anesthetized. Rat was placed in a supine position onto the stereotactic apparatus (DW-2000, Chengdu Techman Software Co., Ltd, China). The rat’s head was fully stabilized by inserting the ear bars into the ear canal. Then the rat’s nose was latched onto the nose bar (15). The bones of the nose are delicate and the nose bar need not be tight. An ophthalmic ointment was preoperatively applied to the rat’s eyes to prevent corneal dehydration. A heating pad was placed under the rat to maintain its body temperature throughout the procedure. In performing the operations depicted below, it’s necessary to employ a binocular surgical microscope at 10× magnification equipped with adequate lights (YH-X-4A, Zhengjiang Yihua Optical Instrument Co., Ltd, China). The rat’s mouth was maximally opened with a laryngoscope resembling device made by curved stainless steel sheet to provide a best view of the epiglottis. Then the appropriate tube was inserted from the glottis into the trachea to complete endotracheal intubation. If necessary, lidocaine (200 μl, 5 ml: 0.1 g) was loaded into the syringe (1 ml) to locally anesthetize the glottis to better complete endotracheal intubation (16).

### Surgical Procedure

The rat’s mouth was opened by custom-made elastic retractors to fix the incisor and tongue. The soft palate was retracted laterally using the other elastic retractors. The oral cavity of the rat was thoroughly sterilized with povidone-iodine (0.5%) in a circular motion. A 0.5 cm longitudinal midline incision of the soft palate was made using an electrotome. The incision should be made to suit for exposing the pituitary gland, making the position of the pituitary more precise. The nasopharyngeal mucoperiosteum covering the sphenoid bone was gently wiped with a cotton swab. When mucoperiosteum was removed, the blood vessel appears as a faint blue line. Then the bone of skull base in front of the blood vessel was drilled with a low speed dental drill of 2 mm diameter. Drilling continues until a thin shelf of bone remains over the pituitary. The remaining bone fragments on the surface of the pituitary gland were removed by a little hook and microforceps. Bleeding was stopped in most instances by temporarily occluding the hole with a surgical sponge or cotton swab. Oral cavity was cleaned with 0.9% saline to keep a clear vision throughout the operation. The pituitary body was identified with the slight pink color. Lentivirus were injected into the pituitary to verify the accurate location of the pituitary gland. Then AAV2/Retro or PRV 152 labeled with EGFP were separately injected into the anterior and posterior pituitary to explore the innervation of the pituitary gland.

### Virus Injection

Injections of 1 μl lentivirus (U6-MCS-Ubi-EGFP, 4×10^8^ TU/ml, constructed by the Genechem Company, Genechem, Shanghai, China), 500 nl AAV2/Retro (pAAV-SYN-MCS-EGFP-3FLAG, 2.4×10^12^ TU/ml, purchased from Obio Technology Co., Ltd, Shanghai, China), or 500 nl PRV 152 (provided by Wuhan Institute of Virology, Chinese Academy of Sciences) were infused through standard glass capillaries, which have an outer diameter/inner diameter (OD/ID) of 1 mm/0.58 mm (Sutter Instrument), driven by a CellTram vario microinjection apparatus (Eppenddorf, Germany), respectively. The injection needles needed to be grinded with a micro grinder (NARISHIGE EG-400, Japan). The difference between the anterior pituitary and posterior pituitary was identified according to the angle and depth during the injection. The needle remained for 5 min following infusion to facilitate diffusion before the needle was slowly removed. Bone wax was applied to fill the drilled hole to protect the pituitary after injection. The soft palate incision was closed with nonabsorbable surgical suture (7-0) after disinfection, and then the rat was placed on a heating pad for recovery and monitored until recovery from anesthesia. Animals were returned to their home cages after recovery from anesthesia and becoming active. If necessary, rats received 0.9% saline to prevent dehydration and additional analgesics (Ketoprofen, 5 mg/kg) for further 2 days.

### Body Weight Measurement

Body weight of rats was measured every morning between 8:00 and 9:00 a.m. during the experimental period.

### Histology

Rats were sacrificed after virus injection for histology. Rats were euthanized with an overdose of pentobarbital sodium (35 mg/kg, i.p.) and transcardially perfused with phosphate buffered saline (PBS) for 5 min. Following PBS flush, brain and pituitary were fixed by perfusing with 4% paraformaldehyde (PFA) in 0.1 M PBS. Brain and pituitary were removed and post-fixed in 4% PFA at 4°C for 24 hr. Then brain and pituitary were transferred to 30% sucrose solution in PBS at 4°C for 2 days. Coronal sections from the sucrose-saturated brain and pituitary were cut at 50 μm on a cryostat (Leica CM1950, Germany). Cryosections were washed with PBS and coverslips and image sections were captured on a laser scanning confocal microscope (Leica TCS SP8, Germany) using a 20× oil objective.

## Results

### The Protocol of Virus Injection Into the Pituitary Gland

**Figure 1:**
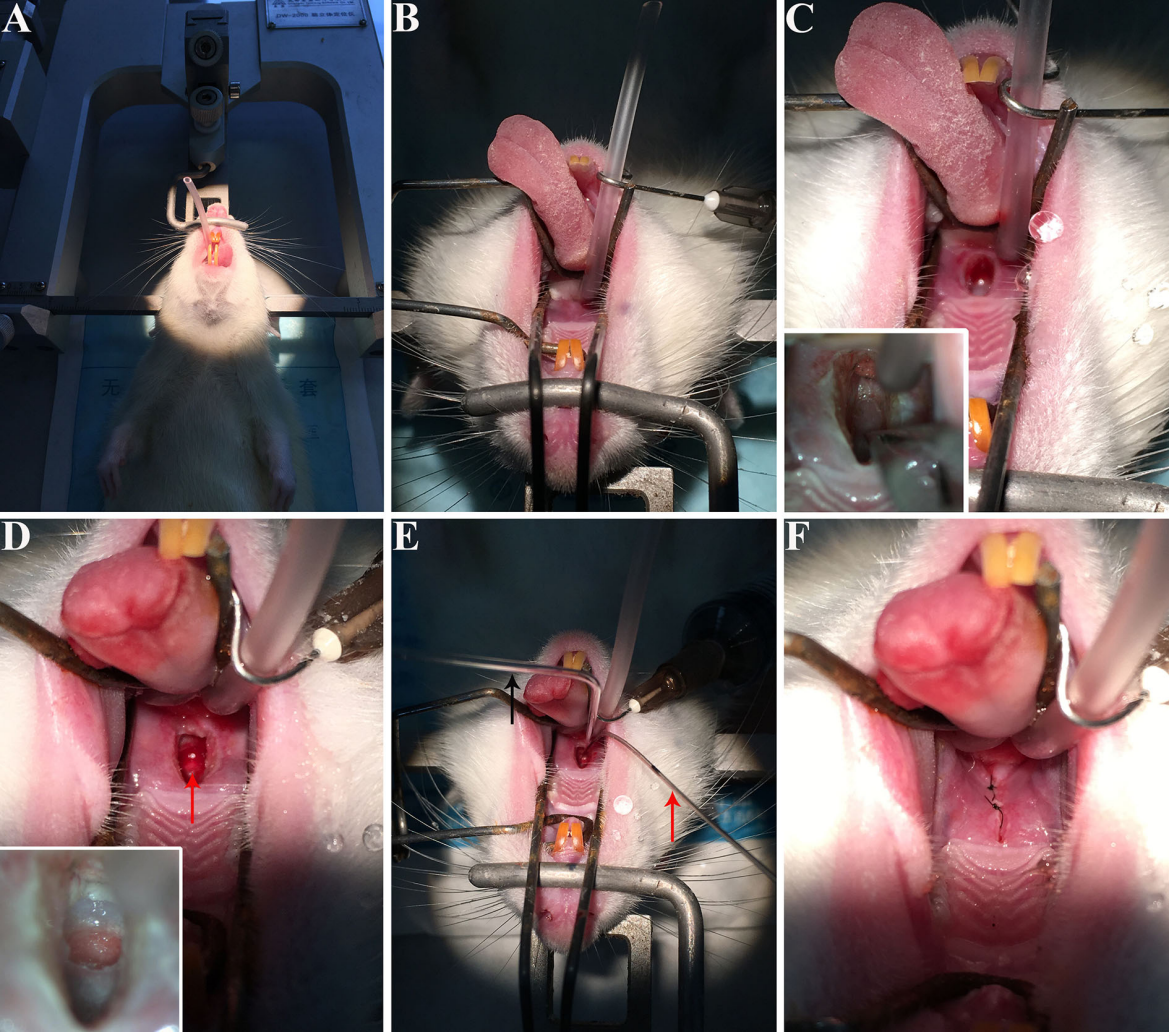
The procedure of virus injection into rat pituitary gland. **(A)** Rat was anesthetized and mounted on a stereotactic apparatus, and then we performed endotracheal intubation. **(B)** The oral cavity of the rat was opened and fixed by custom-made elastic and retracted devices. **(C)** A tiny incision was made into the soft palate. The lower left corner of figure C shows the bone of skull base in front of the blood vessel, drilled with a dental drill. **(D)** The red arrow showed the pituitary body identified by the slight pink color. **(E)** Virus was injected into the pituitary gland. The black arrow shows the injection needles and the red arrow indicates the suction device left in the oral cavity to keep suction to provide a clear vision during injection. **(F)** The incision was closed with surgical suture. These photos were taken by iPhone 6s.

**Figure 2:**
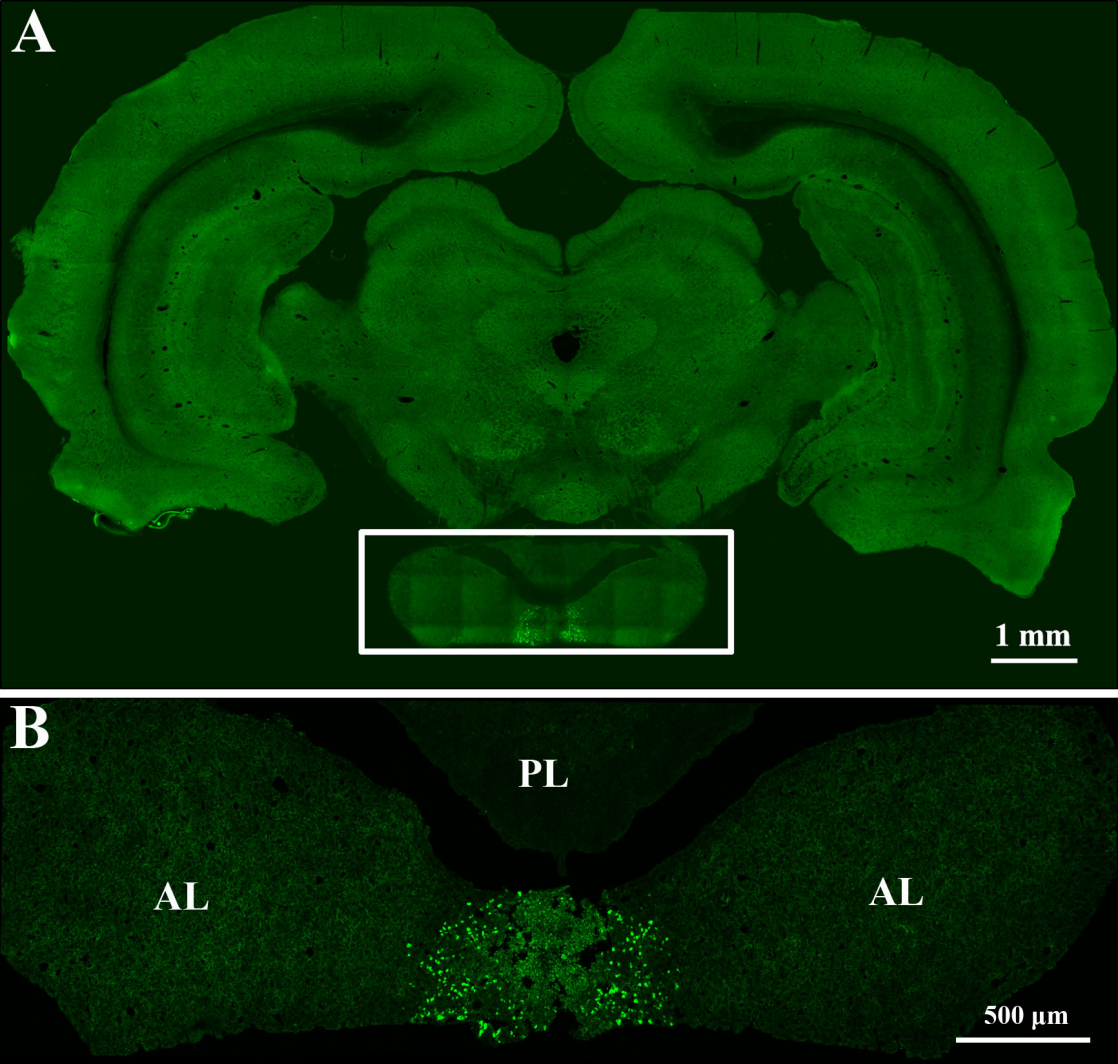
Lentivirus expressing EGFP was accurately injected into the pituitary gland. **(A)** Rat brain coronal slice. The boxed area shows the pituitary gland. Scale bar, 1 mm. **(B)** Magnified pituitary gland in figure A. AL: anterior lobe, PL: posterior lobe. Scale bar, 500 μm.

**Figure 3:**
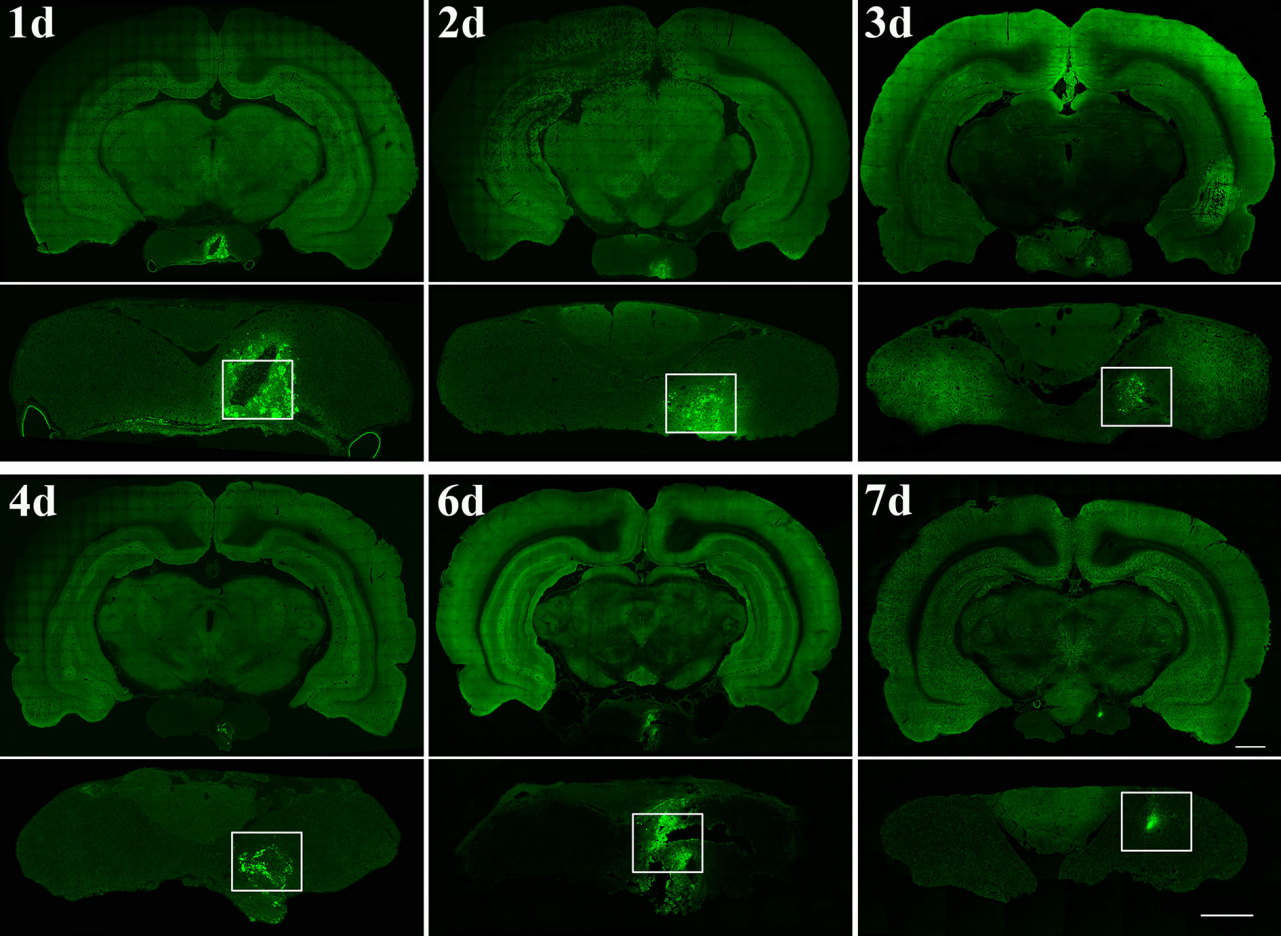
Time points after PRV 152 injection into the anterior lobe. Rats were perfused at 24 hr (day 1, n=3 rat), 48 hr (day 2, n=3 rat), 72 hr (day 3, n=4 rat), 96 hr (day 4, n=4 rat), 144 hr (day 6, n=4 rat), and 168 hr (day 7, n=3 rat) after PRV 152 injection, respectively. The white box indicated the virus injection area of the anterior pituitary. Coronal sections. Scale bar, 1 mm and 500 μm.

**Figure 4:**
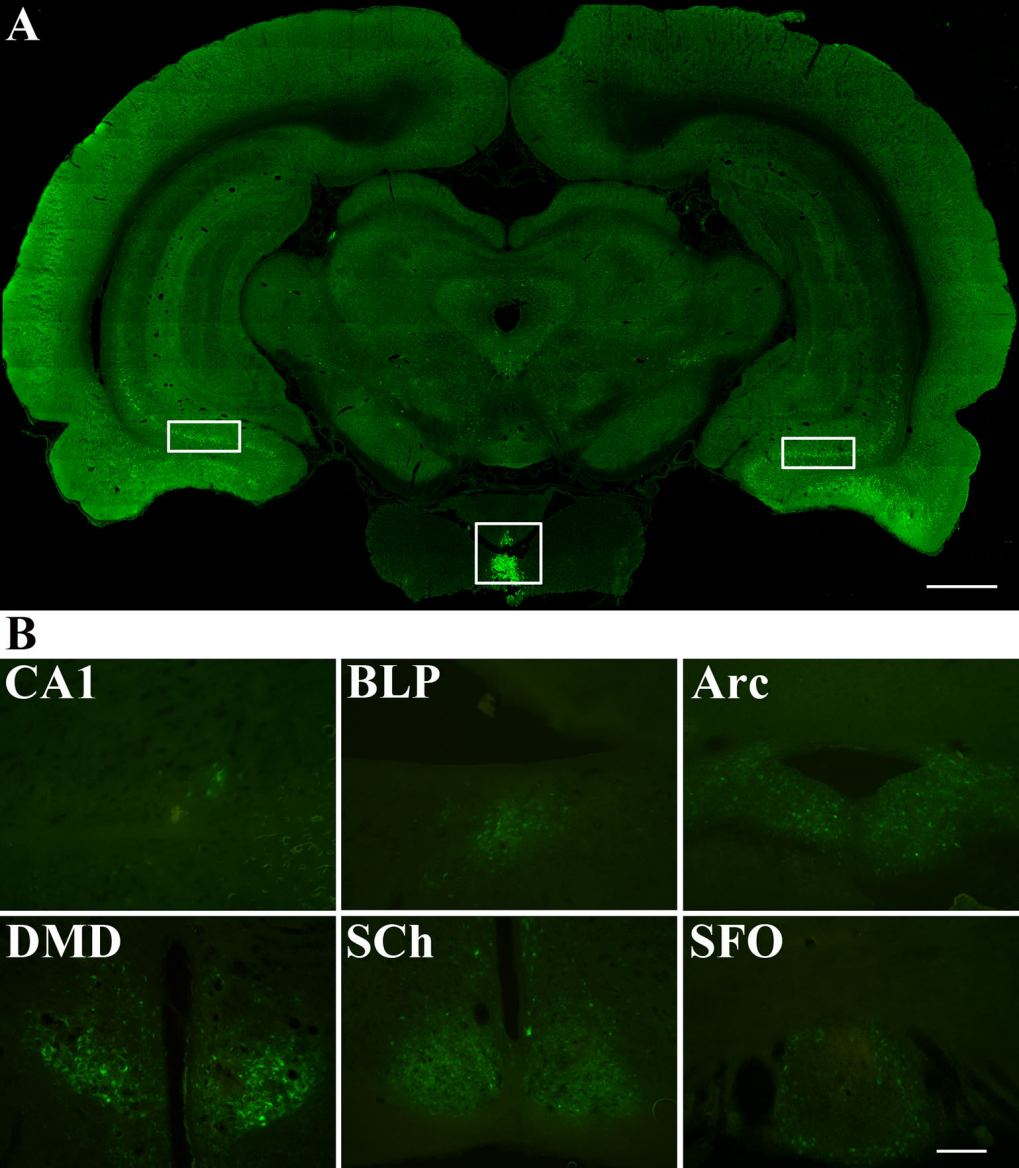
Distribution of PRV 152 infected cells throughout the brain areas that are directly or indirectly associated with the posterior lobe. **(A)** PRV 152 was injected into the posterior pituitary for 96 hr (day 4, n=5). The white square box indicated the virus injection area of posterior lobe. The white rectangular box indicated the distribution of PRV infected cells in the brain of the rat. **(B)** PRV-EGFP positive cells appeared at 96 hr are indicated in the brain region including the field CA1 of hippocampus, basolateral amygdaloid nucleus, posterior part (BLP), arcuate hypothalamic nucleus (Arc), dorsomedial hypothalamic nucleus, dorsal part (DMD), suprachiasmatic nucleus (SCh), and subfornical organ (SFO). Coronal sections. Scale bar, 1 mm and 200 μm.

The method described above involves the following major steps: (1) placing the rat in a supine position onto the stereotactic apparatus (**Figure 1A**), (2) opening the rat’s oral cavity and the soft palate with the custom-made elastic retractor devices (**Figure 1B**), (3) making a precise incision into the soft palate (**Figure 1C**), (4) drilling the bone at the base of skull to expose the pituitary gland (**Figure 1D**), (5) injecting viral tracer into the pituitary gland (**Figure 1E**), and (6) suturing the incision with surgical suture (**Figure 1F**). This protocol provides a technique can be used for hypophysectomy and pituitary injection, allowing the function of the pituitary gland in the endocrine system and in the CNS to be explored.

### The Innervation of the Anterior Lobe With AAV2/Retro or PRV 152 Injection

First, to verify that the pituitary gland can be accurately located by the method described above, we injected lentivirus expressing EGFP into the pituitary gland (**Figure 2**). Then, AAV2/Retro and PRV 152 were injected to investigate the innervation of the anterior pituitary according to the above method. Injection of AAV2/Retro into the anterior lobe was successful, but no green fluorescence was detected by fluorescence microscopy in frozen sections cut from any brain areas or nucleus across the whole brain of rats (data not shown). Next, PRV 152 was injected into the anterior pituitary. Rats were kept alive for between 1 and 18 days after virus injection and sacrificed within this period on a daily basis. We successfully injected PRV 152 expressing EGFP into the anterior lobe at different time points, as shown in **Figure 3**. Within the 1–18 days survival range, there was no green fluorescence detected in frozen sections cut from any brain area or nucleus across the whole brain. Likewise, at 11, 12, 17, and 18 days post-injection, there was no fluorescence detected in the anterior pituitary (data not shown). We injected the same volume of PRV 152 into the anterior pituitary and found that over time post-injection, expression of EGFP decreased and could not be detected after 11, 12, 17, or 18 days. Taken together, these results indicated that there is no direct innervation of anterior pituitary in the rat brain, although it does contain a number of distributed nerve fibers.

### The Innervation of the Posterior Lobe With PRV 152 Injection

**Figure 4** presented the distribution of PRV 152 retrograde cell body labeling through the brain regions that are directly or indirectly linked to the posterior pituitary (**Figure 4A**). These brain regions that PRV 152 infected included the field CA1 of hippocampus, basolateral amygdaloid nucleus, posterior part (BLP), arcuate hypothalamic nucleus (Arc), dorsomedial hypothalamic nucleus, dorsal part (DMD), suprachiasmatic nucleus (SCh), and subfornical organ (SFO) (**Figure 4B**).

## Discussion

Multiple attempts have been made to identify the nerve fibers in the mammalian anterior pituitary over the past couple of decades. It has been demonstrated that the SP-, CGRP-, and galanin-LI nerve fibers are present in the pars distalis of the anterior pituitary using light and electron microscopy combined with immunocytochemistry. Therefore, it is worth exploring the origin of the nerve fibers that innervate the adenohypophysis *via* retrograde transsynaptic tracing based on injection of PRV 152.

The pituitary gland can be exposed using different methods, including those that rely on the transauricular or the parapharyngeal routes. However, these routes have some shortcomings in terms of exploring the nerve fibers that innervate the pituitary gland as they are unable to achieve direct and accurate injection of the pituitary gland. This means that any virus that leaks from the intended injection site will affect the assessment of pituitary innervation and prevent first-order neurons projecting to the target from being reliably confirmed. The present study provides a detailed description of a surgical technique for accessing the pituitary gland in the rat and injecting virus into the anterior or posterior pituitary. It is intended to facilitate researchers to perform this procedure and determine the innervation of hypophysis in the rat. After elucidating the innervation of the pituitary gland, optogenetics or chemogenetics technique can be used to manipulate specific neurons, and further explore the function of the pituitary gland. Transsphenoidal hypophysectomy has been applied for pituitary tumors treatment in dogs and cats (17). For large mammals, such as dog, cat, and rabbit, the transsphenoidal route provides convenient access to the pituitary gland because of the greater size of their oral cavities. However, this operation is not easy in rats and the viral tracer needs to be successfully injected into the very small space of the oral cavity. As in rats, in humans the pituitary glands are located at the base of the brain. Therefore, accessing the pituitary glands *via* the transphenoidal route requires the position of the sella turcica of the sphenoid bone to be accurately determined, followed by opening the sellar floor and stripping the dura. Therefore, thanssphenoidal surgery is an effective technique for pituitary tumors and other intrasellar neoplasms resection (18). A marked and rapid increase in the application of endoscopic transsphenoidal surgery and a decline in microscopic procedures for pituitary neoplasms have been reported (19). In addition to illustrating the extensive application of this method to pituitary tumor removal, these reports also provide us with some points to attend with regard to pituitary exposure and virus injection in the rat.

The protocol for pituitary gland injection described here contains the following critical steps to bear in mind. First, the rat should be endotracheally intubated to prevent death from asphyxiation in the supine position on the stereotaxic apparatus. A surgical microscope with good magnification is also essential to accurately identify the location of the pituitary gland. Second, the incision into the soft palate should be made in a way that exposes the pituitary gland. This allows the position of the pituitary gland to be more precisely located based on the anatomical features of the occipito-sphenoidal suture line at the base of the skull. It is also important to pay attention to the strength with which the hole is drilled to avoid excessive force that could damage the pituitary gland. Third, the suction device should be placed in the oral cavity to maintain suction so that visibility is not blocked during the injection of the viral tracer. Finally, the soft palate incision should be sutured to prevent intestinal distention, which could kill the rat. Rats should be supervised more frequently and closely during recovery (based on their behavior and feed and water consumption) and provided with soft food after surgery. The whole process from anesthesia to pituitary gland injection can be completed in as little as 1 hr, if performed with a high degree of efficiency. The surgeon who becomes skilled in this procedure can easily complete the operation with a high degree of success. The method described above has been performed on more than 40 rats in our experiments, with no postoperative death and no signs of inactivity in rats during the postoperative period. Additionally, we have found no statistically significant loss of body weight in rats between the preoperative period and the eventual sacrifice of the animal. In our experiment, survival times after PRV 152 injection in rats range from 1 to 18 days, after which the animals were perfused. We further found that the soft palate incision was generally recuperated by day 4. Despite the advantages of the approach described above, for those new to the technique, it can be difficult to accurately confirm the location of the pituitary gland, and the inaccurate bone drilling can lead to severe hemorrhage. Crucially, the custom-made devices used for the operation cannot be purchased and need to be made by the operator. We expect that more people will try to improve this approach because of its obvious potential for application to hypophysectomy or pituitary injection.

PRV 152 is known for its neurotropic nature and property to spread retrogradely within synaptically linked neurons, and it has been widely used in numerous mammalian tracing studies. The application of PRV as a transneuronal retrograde tracer depends on its ability to invade chains of hierarchically connected neurons, and then to replicate and spread to synaptically linked neurons (20, 21). Therefore, the use of PRV as a neural tracer makes it a powerful tool for delineating central circuits. In the present study, we injected PRV 152 into the anterior pituitary of the rat to explore the origins of nerve fibers innervating this structure using the method described above. PRV 152 was successfully injected into the anterior pituitary of rats, which were then sacrificed on days 1–18 post-injection. The brain and pituitary gland were continuously sectioned, but we found no EGFP expression in any brain regions. The volume of the viral infection within the anterior pituitary was as much as 1 mm^3^ approximately. With increasing survival times post-injection, the expression level of PRV 152 in the anterior pituitary gland associated with the same injection volume gradually decreased until the virus had been cleared completely at day 18, and no EGFP could be detected. These results demonstrate that the anterior pituitary of the rat is not innervated other than by a few autonomic nerve fibers. It’s acknowledged that anterior pituitary in the rat is regulated by transmission from the hypothalamus *via* the portal system (22), and that the adenosine cells of the anterior lobe receives humoral regulation. The particular functions of these nerve fibers in the anterior pituitary need to be further explored and much remains to be elucidated. Furthermore, we successfully injected PRV 152 into the posterior lobe in rats. Following the injection of viral tracer into the posterior pituitary, after 4 days, labelled neurons were identified in the these brain regions: paraventricular hypothalamic nucleus (PVH), arcuate hypothalamic nucleus (Arc), basolateral amygdaloid nucleus, posterior part (BLP), periventricular hypothalamic nucleus (Pe), suprachiasmatic nucleus (SCh), and subfornical organ (SFO) (the figures of PVH and Pe are not shown in Results). These results are in accordance with previous reports in that the labelled neurons derived from Arc, PVH, and Pe could be responsible for dopaminergic or glutamatergic innervation of the posterior pituitary (23, 24). However, the type of labeled neurons in these brain regions in the present study still need to be identified. After longer post-injection intervals, neurons in SCh and SFO also expressed EGFP as they became infected after transsynaptic uptake and retrograde transport from synaptically connected neurons.

On the basis of this discussion, we have shown that the operation presented here provides a promising method for performing hypophysectomy or pituitary injection. This allows the function or the innervation of the pituitary gland to be clarified and offers crucial advantages, especially given the evidence that pituitary gland is a fundamental regulator of the endocrine network and the CNS.

## Data Availability Statement

The original contributions presented in the study are included in the article/supplementary materials. Further inquiries can be directed to the corresponding author.

## Ethics Statement

The animal study was reviewed and approved by The Animal Care and Use Committee of Hubei University of Medicine (Hubei, China).

## Author Contributions

CK and WJ conceived the project and designed the study. XL, SS, HZ, and YL performed the experimental studies. XX, YG, DS, and ZY acquired and analyzed the data. XL prepared and revised the manuscript under the guidance of CK. All authors contributed to the article and approved the submitted version.

## Funding

This work was supported by the National Natural Science Foundation of China (81971060), the Natural Science Foundation of Hubei Provincial Department of Education (B2018116), and the Scientific and Technological Project of Shiyan City of Hubei Province (19Y39, 19Y41).

## Conflict of Interest

The authors declare that the research was conducted in the absence of any commercial or financial relationships that could be constructed as a potential conflict of interest.
